# Supplementary material for: A Simplified Genomic Profiling Approach Predicts Outcome in Metastatic Colorectal Cancer
Source: Cancers (Basel). 2019 Jan 27;11(2):147. doi: 10.3390/cancers11020147 (PMC6406354; doi:10.3390/cancers11020147)
Supplement: Supplementary file 1 [file cancers-11-00147-s001.pdf]

Supplementary Materials: A Simplified Genomic Profiling Approach Predicts outcome in Metastatic Colorectal Cancer

Carlo Capalbo, Francesca Belardinilli, Domenico Raimondo, Edoardo Milanetti, Umberto Malapelle, Pasquale Pisapia, Valentina Magri, Alessandra Prete, Silvia Pecorari, Mariarosaria Colella, Anna Coppa, Caterina Bonfiglio, Arianna Nicolussi, Virginia Valentini, Alessandra Tessitore, Beatrice Cardinali, Marialaura Petroni, Paola Infante, Matteo Santoni, Marco Filetti, Valeria Colicchia, Paola Paci, Silvia Mezi, Flavia Longo, Enrico Cortesi, Paolo Marchetti, Giancarlo Troncone, Diana Bellavia, Gianluca Canettieri and Giuseppe Giannini

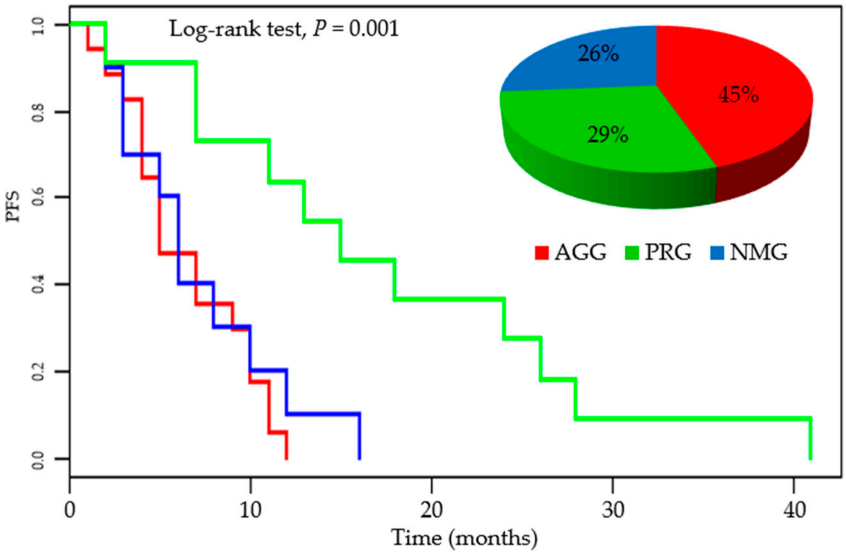

Groups	N° pz	Median PFS (months)
Overall population	38	7
AGG	17	5
PRG	11	15
NMG	10	6

Comparisons	*p* value
AGG *vs* PRG	<0.001
AGG *vs* NMG	0.555
PRG *vs* NMG	0.006

Figure S1. Kaplan–Meier plot showing the impact of PRG (green), AGG (red) and NMG (blue) group stratification on progression free survival (PFS) in the KRAS WT mCRCs.
